# Applying ecological site concepts and state‐and‐transition models to a grazed riparian rangeland

**DOI:** 10.1002/ece3.4057

**Published:** 2018-04-19

**Authors:** Felix Ratcliff, James Bartolome, Luke Macaulay, Sheri Spiegal, Michael D. White

**Affiliations:** ^1^ Department of Environmental Science Policy and Management University of California, Berkeley Berkeley California; ^2^ USDA‐ARS‐Jornada Experimental Range Las Cruces New Mexico; ^3^ Tejon Ranch Conservancy Frazier Park California

**Keywords:** CART analysis, ecological site descriptions, grazing management, hierarchical cluster analysis, riparian classification

## Abstract

Ecological sites and state‐and‐transition models are useful tools for generating and testing hypotheses about drivers of vegetation composition in rangeland systems. These models have been widely implemented in upland rangelands, but comparatively, little attention has been given to developing ecological site concepts for rangeland riparian areas, and additional environmental criteria may be necessary to classify riparian ecological sites. Between 2013 and 2016, fifteen study reaches on five creeks were studied at Tejon Ranch in southern California. Data were collected to describe the relationship between riparian vegetation composition, environmental variables, and livestock management; and to explore the utility of ecological sites and state‐and‐transition models for describing riparian vegetation communities and for creating hypotheses about drivers of vegetation change. Hierarchical cluster analysis was used to classify the environmental and vegetation data (15 stream reaches × 4 years) into two ecological sites and eight community phases that comprised three vegetation states. Classification and regression tree (CART) analysis was used to determine the influence of abiotic site variables, annual precipitation, and cattle activity on vegetation clusters. Channel slope explained the greatest amount of variation in vegetation clusters; however, soil texture, geology, watershed size, and elevation were also selected as important predictors of vegetation composition. The classification tree built with this limited set of abiotic predictor variables explained 90% of the observed vegetation clusters. Cattle grazing and annual precipitation were not linked to qualitative differences in vegetation. Abiotic variables explained almost all of the observed riparian vegetation dynamics—and the divisions in the CART analysis corresponded roughly to the ecological sites—suggesting that ecological sites are well‐suited for understanding and predicting change in this highly variable system. These findings support continued development of riparian ecological site concepts and state‐and‐transition models to aid decision making for conservation and management of rangeland riparian areas.

## 1 INTRODUCTION

Riparian areas threading through upland rangelands boost landscape‐level biodiversity (Sabo et al., 2005), filter water (Tate, Atwill, Bartolome, & Nader, 2006), and provide other valuable ecosystem services (George, Jackson, Boyd, & Tate, 2011). They also provide forage and water for livestock, which tends to congregate these areas, potentially degrading riparian resources (Belsky, Matzke, & Uselman, 1999; Kauffman & Krueger, 1984). Accordingly, efforts to improve the outcomes of riparian management are common, but the highly variable and site‐specific responses of rangeland riparian zones can complicate managers’ ability to make reliable predictions about the effects of management (George et al., 2011).

Ecological site descriptions and state‐and‐transition models are currently regarded as useful organizing frameworks for understanding and predicting the patterns and processes on rangelands (Spiegal et al., 2016; Sayre, 2017). These models have been extensively developed for upland rangelands in the United States, but only recently has attention been given to developing them for riparian systems (Stringham & Repp, 2010).

Major determinants of riparian rangeland vegetation composition include fluvial processes and their controls on channel geomorphology (McBride & Strahan, 1984; Stella, Battles, McBride, & Orr, 2010), depth to water table and soil moisture dynamics (Stringham, Krueger, & Thomas, 2001), inundation frequency (Sankey, Ralston, Grams, Schmidt, & Cagney, 2015), annual fluctuations in precipitation (Lunt, Jansen, Binns, & Kenny, 2007), and flood disturbance regimes (Campbell & Green, 1968). As a result of frequent disturbances and spatial heterogeneity, vegetation will likely never reach “climax” stages (Campbell & Green, 1968), and biotic drivers such as cattle grazing may have limited effects on vegetation composition (Lunt et al., 2007). Nevertheless, cattle tend to congregate in riparian areas and can have exaggerated effects on these systems (Kauffman & Krueger, 1984), and managers need models that consider the role of abiotic disturbances, livestock management, and site potential.

Rangelands in Mediterranean‐type climates, which are predictably mesic in the winter and xeric in the summer, have distinct flora, fauna and unique management systems, conservation challenges, and threats (Bartolome et al., 2014; Perevolotsky & Seligman, 1998). Riparian systems in Mediterranean‐type regions have high interannual and intra‐annual weather variation coupled with a “flashy” hydrology produced during the relatively short wet season, creating periodic fluvial disturbances and drought which structure biological communities (Gasith & Resh, 1999). Models that consider these abiotic perturbations may be necessary to describe vegetation dynamics in Mediterranean‐type riparian systems.

State‐and‐transition models are usually represented by box and arrow diagrams and descriptive text that catalogs all the known vegetation states (boxes) and transitions between states (arrows) for a given site. They were developed to model nonlinear vegetation dynamics in rangeland systems (Westoby, Walker, & Noy‐Meir, 1989) and are a useful tool for communicating vegetation dynamics to managers. Ecological sites describe divisions of the landscape with similar environmental characteristics that support the same range of states and transitions (Spiegal, Larios, Bartolome, & Suding, 2014). Given the variable nature of rangeland riparian sites, ecological site descriptions and state‐and‐transition models may be the optimal framework for cataloguing and making predictions about their ecology (Stringham & Repp, 2010)—but more information is needed about how to best classify ecological sites, states, and phases for rangeland riparian areas.

Upland sites are largely classified based on soils, climate, and landscape position, which are relatively stable over timescales relevant to management (Caudle, DiBenedetto, Karl, Sanchez, & Talbot, 2013). These factors are probably not sufficient to describe differences in rangeland riparian sites, because riparian sites are also influenced by differences in fluvial processes, channel geomorphology, and hydrologic cycles between sites (Stringham & Repp, 2010).

Processes governing temporal variation within riparian ecological sites differ somewhat from those in uplands as well. In addition to climatic and management drivers associated with interannual variation in uplands, fluvial processes and soil–water characteristics may drive temporal variation in vegetation composition (Stringham & Repp, 2010; Stringham et al., 2001). Linking characteristics of channel geomorphology, soils, and hydrologic properties to differences in riparian vegetation states is necessary to help pair riparian ecological site descriptions with state‐and‐transition models.

Given their value to conservation and management, it is important to understand riparian systems in rangelands so that their management can be improved. This study addresses the following research questions:

1. Can ecological sites and state‐and‐transition models be used to describe riparian vegetation assemblages and develop hypotheses about their relationships to environmental and management (i.e., cattle grazing) variables?
2. In addition to parameters typically used in upland ecological site classification, what new parameters are needed to classify riparian ecological sites in a Mediterranean‐type system?

## 2 MATERIALS AND METHODS

### 2.1 Study site

Tejon Ranch, located in southern California, contains 97,124 hectares of conserved lands that are jointly managed by the Tejon Ranch Company, Tejon Ranch Conservancy, and two grazing lessees. Cattle grazing is the most widespread land management practice affecting riparian areas on the ranch. The Ranch encompasses areas of California's San Joaquin Valley, Sierra Nevada Mountains, Mojave Desert, Tehachapi Mountains, and South Coast Ranges. This study is limited to major streams with well‐developed woody vegetation, in the San Joaquin Valley portion of the ranch. Despite large‐scale conversion of riparian forests in California's Central Valley, these areas provide a wide array of ecosystem services (Vaghti & Greco, 2007); due to its extent and management history, Tejon Ranch provides an ideal location to study a relatively intact network of Central Valley riparian forests.

**Figure 1:**
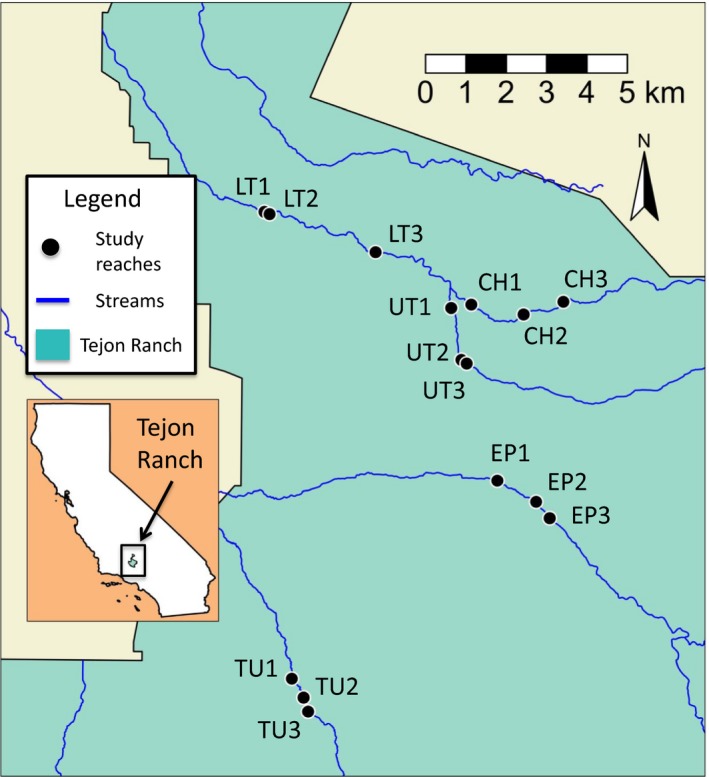
Map of study reaches on Tejon Ranch

Five creek segments were selected for study within the area of interest: Chanac Creek (CH), El Paso Creek (EP), Lower Tejon Creek (LT), Tunis Creek (TU), and Upper Tejon Creek (UT)—hereafter referred to as “creek segments.” Within each of these creek segments, three locations were selected randomly within areas with woody vegetation for a total of 15 study reaches—hereafter referred to as “study reaches” (Figure 1). In the winter of 2014–2015, one study reach on each stream segment was randomly chosen to receive a cattle exclosure. The exclosures were in place for the remainder of the study. Reaches that received exclosures were CH2, EP3, LT1, TU2, and UT3.

Although somewhat drier than the “true” Mediterranean climate (Aschmann, 1984), the study area is in a Mediterranean‐type region of California, with hot dry summers and cool wet winters. Mean annual precipitation is 21 cm. Eighty‐nine percent of this falls between November and April (http://ipm.ucanr.edu/WEATHER/wxactstnames.html). Mean maximum summer daily temperatures are between 32 and 35°C, and mean minimum summer temperatures are between 15 and 19°C. Mean maximum winter daily temperatures are between 15 and 21°C, and mean minimum daily winter temperatures are between 3 and 8°C. The 4 years encompassed by this study had below‐average rainfall. Between 2012 and 2015, the average annual precipitation was only 15.7 cm. The 2015–2016 rain‐year had approximately average rainfall (20 cm).

### 2.2 Sampling abiotic site factors

Fourteen variables were used to classify the 15 study reaches into ecological sites (Table 1). These include remotely sensed values measured in a geographical information system, such as elevation, slope, sinuosity, watershed size, and geology (Table 1). Measurements of stream geomorphology were made in the field using a total station. Soil samples were collected in the field in 2013 and analyzed for soil texture using the hygrometer method at the UC Davis Analytical Laboratory.

**Table 1:** Variables used in the ecological site cluster analysis

Variable	Source	Description
Elevation (m)	Field	Measured in a geographical information system (GIS) from points taken using a GPS
Slope (500 m)	GIS (10 m spatial resolution Digital Elevation Model [DEM])	Slope along a 500 m portion of the creek centered at the plot center (NRCS National Cartography and Geospatial Center, 2010)
Slope (thalweg)	Field	Slope of the thalweg on the study reach taken from long profile field measurements
Sinuosity (500 m)	GIS	Distance along creek divided by Euclidian distance between endpoints for 500 m of creek
Sinuosity (thalweg)	Field	Distance along creek divided by Euclidian distance between endpoints of the long profile measured in study reach
Watershed Size	GIS (10 m DEM)	Area of watershed contributing to stream at study reach (NRCS National Cartography and Geospatial Center 2010)
Geology	GIS	Mapped geology at study reach. (Dibblee, 2005, 2008a,b)
Dominant Upstream Geology	GIS	Most common geology mapped along stream between study reach and headwaters. (Dibblee, 2005, 2008a,b)
Width:Depth Ratio	Field	Width of stream channel divided by depth of channel. Calculated from channel cross‐sections
Entrenchment Ratio	Field	Width of flood‐prone area divided by width of channel. Calculated from channel cross‐sections
Greenline Height Above Thalweg	Field	Average height of greenline above thalweg in the study reach.
Sand (%)	Field	Percent sand in composite soil sample along greenline
Silt (%)	Field	Percent silt in composite soil sample along greenline
Clay (%)	Field	Percent clay in composite soil sample along greenline

### 2.3 Sampling vegetation

Vegetation was sampled annually at each of the study reaches in late May and early June of 2013 through 2016. A “greenline” transect followed the toe of the creek bank and sampled vegetation growing near the water's edge. Winward (2000) recommends sampling greenline vegetation at the top of bank; however, we sampled the greenline at the toe of the bank because the herbaceous vegetation at the top of bank was typically composed of the same annual grass species that dominate the adjacent uplands. In order to sample the herbaceous species composition most influenced by the stream, we needed to sample in the wetter soils found at the toe of the bank. Shrubs and trees were recorded in the sampling transects regardless of position.

Greenline vegetation composition was measured along 50 m of the 15 study reaches in three different strata: herbaceous, shrub, and tree. The starting point for each transect was the randomly assigned center point of the study reach. Herbaceous vegetation was measured with a line‐point intercept transect. Each half‐meter along the transect tape, the first plant hit within one meter above the ground was recorded. A line‐intercept transect was used to record the linear distance of shrubs and trees overhanging the greenline transect. Any plant (regardless of species) overhanging the tape between one and 3 m of height was recorded in the “shrub” category, and any plant overhanging the tape above 3 m in height was recorded in the “tree” category.

### 2.4 Statistical methods

Our analytical approach proceeded as follows: (1) cluster analysis was used to classify stream reaches into ecological sites with respect to their abiotic site factors and (2) to define meaningful vegetation community assemblages (vegetation clusters); (3) Indicator Species Analysis was used to describe the vegetation clusters; (4) the presence of states were evaluated as aggregations of clusters; (5) CART analysis was used to identify the influence of abiotic site factors on vegetation cluster differentiation.

### 2.5 Ecological site cluster analysis

Hierarchical cluster analysis can be used to classify ecological sites based on groupings of key abiotic environmental variables in rangeland uplands (Spiegal et al., 2014). Similarly, it has been used to classify stream reaches from geomorphic and hydrologic measurements of stream channels and is especially useful if applied within a distinct physiographic unit where it can yield objective classifications (Kondolf, Montgomery, Piegay, & Schmitt, 2003). A suite of indicators used in stream classification and those used in upland ecological site classification were combined in a cluster analysis to create the riparian ecological site classification (Table 1).

The ecological site cluster analysis was performed using Gower's distance, which calculates similarity for each variable in the matrix separately (using a method according to the variable type) and is therefore able to analyze both continuous and categorical variables together. The final distance metric is an average of the partial similarities (Borcard, Gillet, & Legendre, 2011). Analysis was performed in R using the packages “vegan” and “cluster” (Maechler, Rousseeuw, Struyf, Hubert, & Hornik, 2015; Oksanen et al., 2015; R Core Team 2016). The cluster dendrogram was pruned using the Mantel test, which compares a matrix of cluster assignments to the original distance matrix used to create the cluster dendrogram. This test is repeated for every possible number of clusters, and the number with the highest Mantel correlation is considered the optimal number of clusters (Borcard et al., 2011).

### 2.6 Vegetation cluster analysis

A cluster analysis was performed on the greenline vegetation cover data to investigate patterns of riparian plant community structure within the 15 study reaches over 4 years. The 60 unique Reach × Year combinations were clustered based on absolute cover of all live plants along transects. Proportional cover data from the shrub and tree layers were generally much higher than data from the herbaceous layer; therefore, herbaceous layer data were square‐root transformed so that the shrub and tree layers did not overly influence the cluster assignments (McCune, Grace, & Urban, 2002). Shrub and Tree cover was not transformed. Similarly, all species occurring on <2 Reach × Years were removed from the analysis so that very rare species did not disproportionately influence the analysis. All species × canopy class combinations were treated as unique species. The cluster analysis was performed using Bray–Curtis distance, which calculates similarity based on species found to be present on study reaches rather than mutual absences (Zuur, Ieno, & Smith, 2007).

Two methods were utilized to prune the cluster dendrogram to the optimal number of clusters. First, a Mantel correlation test was used, as described above. Second, an Indicator Species Analysis was performed, and the number of groups which contained the most significant indicator species was selected (Dufrene & Legendre, 1997; McCune et al., 2002). All statistical analyses were performed in R using packages: vegan, cluster, and indicspecies (De Caceres & Legendre, 2009; Maechler et al., 2015; Oksanen et al., 2015; R Core Team 2016).

### 2.7 Indicator species analysis

In addition to showing the optimal location to prune the cluster dendrogram, the “significant” indicator species show which species best characterize each cluster. Indicator species are those that are common within study reaches of one cluster, and relatively scarce in study reaches of other clusters (Dufrene & Legendre, 1997). Based on these criteria, species are given an indicator value (0–1), and a randomization test is performed to determine the statistical significance of the indicator value. “Significant” indicator species are those with <5% probability of having no difference between groups.

### 2.8 Identifying states, phases, and transitions

The USDA Natural Resources Conservation Service (NRCS) implementation of state‐and‐transition models and ecological site descriptions is largely a “top‐down” process where elements of state‐and‐transition models are drawn and populated by expert opinion and afterward validated with data (Jackson, Bartolome, & Allen‐Diaz, 2002). In contrast, in this study, plant species data are aggregated to build vegetation states from the “ground‐up” with fewer preconceptions about what constitutes a “state.” The NRCS also differentiates between minor, easily reversible changes in vegetation labeled “phase‐shifts” and the more resilient “states” they occur in (Bestelmeyer et al., 2003; Stringham, Krueger, & Shaver, 2003). These distinctions formalize some general aspects of the original state‐and‐transition approach and provide useful categories that can be the basis of testable hypotheses.

In this study, we performed cluster analysis to define meaningful vegetation assemblages. Many of these clusters had similar vegetation structure and functional group composition—thus having similar implications for management—and transitions between some of these clusters would likely occur without threshold dynamics. As a result, the optimal number of clusters from the vegetation cluster analysis was considered vegetation “phases,” not “states.” The more general “states” were defined by considering: potential drivers of spatial and temporal variation (e.g., irreversible geomorphological changes), differences in Bray–Curtis distance between the clusters, and ecological characteristics of the dominant and indicator species of each cluster (e.g., wetland vs. upland plants). The resulting states are still based on the original vegetation cluster dendrogram, but represent a deeper “cut” of the dendrogram with fewer terminal nodes.

The vegetation cluster analysis was performed on data from all 15 study reaches, and the resulting states and phases were subsequently divided into the two ecological sites. This procedure was chosen because (1) it allowed evaluation of how well the ecological sites corresponded to observed differences in vegetation dynamics, and (2) although study reaches are represented by discreet ecological sites, they represent a gradient of site characteristics and are therefore expected to share some vegetation states. Combining data from all study plots showed which states are unique to each ecological site, and which are shared between them.

In our scheme, a “temporal transition” occurs when the state at a study reach moves in species cluster space between years (sensu Spiegal et al., 2014). “Spatial transitions” are evident in cases in which different vegetation clusters occur in different areas within the same ecological site and are differentiated by spatial—instead of inherently temporal—processes (also see Bestelmeyer, Goolsby, & Archer, 2011).

### 2.9 Classification Tree (CART)

A classification tree was built to determine which environmental factors best predict the observed vegetation states and to inform our ecological site classification approach. The response variable (the data to be partitioned) was the clusters from the vegetation cluster analysis, and the independent variables were the environmental variables used in the ecological site cluster analysis, annual precipitation, and grazing treatments (exclosures). A classification tree uses top‐down recursive binary splitting to partition the response data into a tree that optimizes the classification of response variables at each node with respect to each of the predictor variables (James, Daniela, Trevor, & Robert, 2013).

The classification tree was built using the “tree” package in R (Ripley, 2016). The tree was pruned using the function “cv.tree,” which determines the optimal number of terminal nodes by minimizing the deviance in a *K*‐fold cross‐validation (Ripley, 2016). Pruning the CART tree to seven terminal nodes resulted in the lowest deviance in the CART analysis. This resulted in only six of the fourteen abiotic factors being included in the construction of the classification tree (Table 2).

**Table 2:** Mean values per ecological site for each of the environmental attributes used in the ecological site cluster analysis. Geology values list all the geology mapping units in each ecological site

	Ecological site 1	Ecological site 2	Selected by CART model?
Elevation (m)	444	265	Yes
Slope (500 m)	3.1%	1.7%	Yes
Thalweg slope	2.8%	1.0%	No
Sinuosity (500 m)	1.10	1.15	No
Sinuosity of Thalweg	1.20	1.14	No
Watershed size (m^2^)	9.68 × 10^7^	2.82 × 10^8^	Yes
Width: Depth ratio	8.49	9.97	No
Entrenchment ratio	3.39	5.86	No
Greenline height above Thalweg (m)	0.213	0.223	No
Sand (%)	79.5	87.0	Yes
Silt (%)	14.83	7.33	Yes
Clay (%)	5.67	5.67	No
Geology	Qt, gn, hdq^1^	Qa	Yes
Dominant upstream geology	Qt, gn, hdq	Qa	No

Geology abbreviations indicate the following geology map units (Dibblee, 2005, 2008a,b): gn = Gneissic Rocks; hdq = Mafic Intrusive Rock; Qa = Quaternary Alluvium; Qt = Quaternary Terrace Deposits.

## 3 RESULTS

### 3.1 Ecological sites

**Figure 2:**
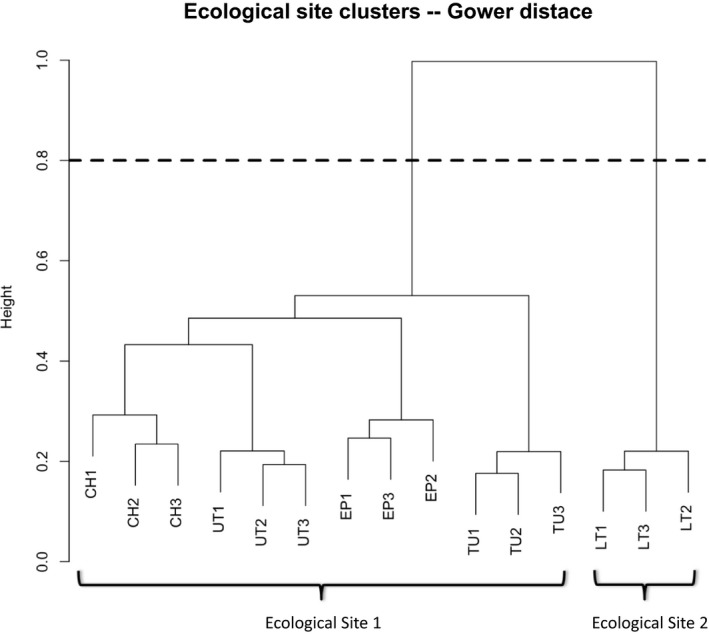
Cluster dendrogram showing the results of the ecological site cluster analysis based on abiotic variables. The more closely their branches are related in the dendrogram, the more similar their environmental attributes are. The letters in the study reach names represent the creeks they are on. CH = Chanac Creek, EP = El Paso Creek, LT = Lower Tejon Creek, TU = Tunis Creek, UT = Upper Tejon Creek. The dotted line shows the optimal location to prune the dendrogram

The Mantel correlation test showed that the optimal number of clusters was 2 (*r* = .644), representing two ecological sites: Lower Tejon Creek and all other study reaches (Figure 2). The *r* value for the next highest correlation (for five clusters) was substantially lower at *r* = .572.

These two ecological sites differ in several regards. Ecological Site 1 is more widespread in the study area and as a result is more variable. Reaches in Ecological Site 1 (all study reaches except those on Lower Tejon Creek) have higher elevations, higher channel slopes, smaller watershed sizes, lower entrenchment ratios, more silt and less sand in the soil, and more diverse geologies and upstream geologies than reaches in Ecological Site 2 (those on Lower Tejon Creek). The variables that do not substantially differ between the two ecological sites are sinuosity, width:depth ratio, greenline height above thalweg, and percent clay in soil (Table 2).

### 3.2 Vegetation states

**Figure 3:**
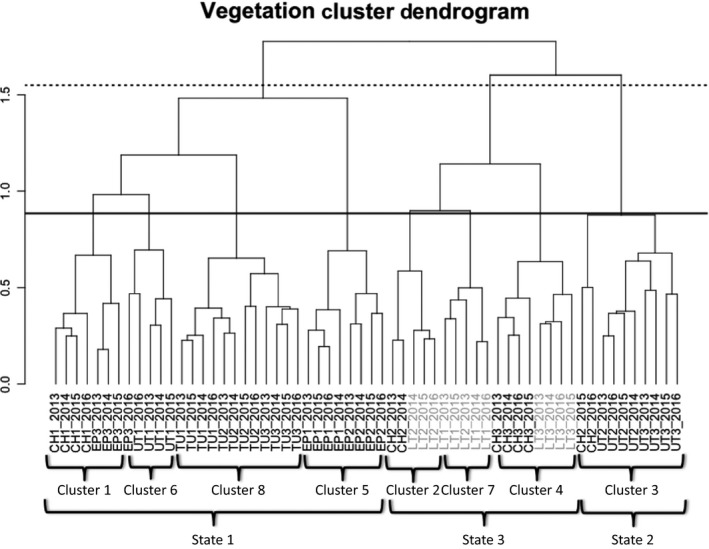
Cluster dendrogram showing the results of the vegetation cluster analysis. The units being clustered are all the Reach × Years. The more closely related branches in the dendrogram have more similar vegetation. The letters in the Reach × Year names represent the creeks they occur on. Reach names in black are in Ecological Site 1; names in gray are in Ecological Site 2. The solid and dotted lines show where to trim the dendrogram to produce eight community phase clusters and three vegetation states, respectively

The vegetation cluster analysis showed that Reach × Years generally clustered most closely with the same reach in other years. The Mantel correlation test pruned the resulting dendrogram to 10 clusters (*r* = .663). However, eight clusters had the most significant indicator species *p*‐values and were therefore selected by indicator species analysis. As the Mantel correlation coefficient was very close between eight and ten clusters, (*r* = .645 and *r* = .663 respectively), eight clusters were selected to represent the vegetation groups (Figure 3).

**Figure 4:**
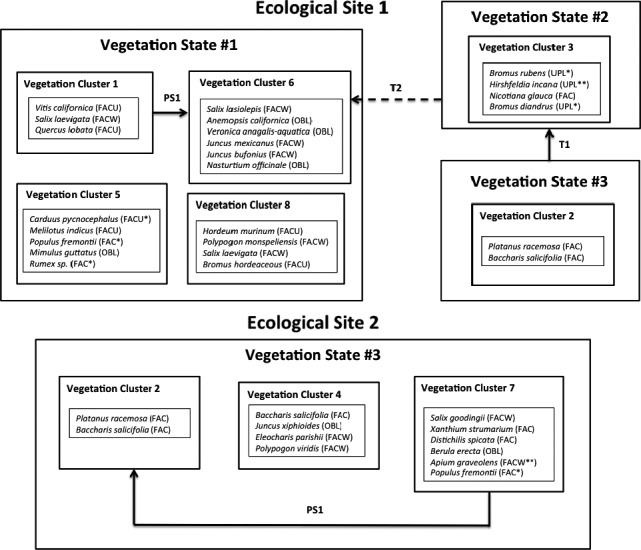
State‐and‐transition diagram for the riparian study reaches. The top diagram shows the states and phases occurring on study reaches in Ecological Site 1, and the bottom diagram shows state and phases occurring on reaches in Ecological Site 2. Species listed in each phase are the significant indicator species for that phase, listed by descending order of indicator value. Solid arrows indicate “temporal transitions” and phase shifts, the dotted arrow shows the only “spatial transition” with a plausible driver. Wetland codes are provided in parentheses after each species name (Lichvar, Banks, Kirchner, & Melvin, 2016). An * indicates that the species is not included in “The National Wetland Plant List” (Lichvar et al., 2016). The wetland status of species with one * is inferred from congeners on the list. Species with two ** do not have congeners on the list, and their wetland status is hypothesized from authors’ field observations. More information on plant species is included in Table S1. Descriptions of the states and transitions are in the text of the Results section

Each of the eight clusters has statistically significant indicator species. All clusters include perennial woody species as indicators, and all clusters except Clusters 1 and 2 include herbaceous species as significant indicator species (Figure 4). Indicator species are always most abundant in the cluster they are assigned to; however, in this analysis, they generally occur in other clusters as well, so their mere presence is not diagnostic of cluster membership. Just five of the 41 indicator species occurred in only one cluster, and five occurred in all eight clusters. Thirty‐five of the 41 indicator species were in the top five species (by cover) for their canopy layer in the cluster they belonged to.

Per methods described previously, three vegetation “states” were defined among the eight vegetation clusters (Figure 3). States 1 and 2 occur exclusively in Ecological Site 1, while State 3 occurs almost exclusively in Ecological Site 2, but has a limited distribution on Ecological Site 1 (Figure 3). These three states represent a deeper “cut” of the dendrogram and also have a high Mantel correlation value (*r* = .61). The three states are:

#### 3.2.1 Vegetation state 1

This state comprises four of the eight vegetation clusters (Clusters 1, 5, 6, and 8) that are closely branched on the cluster dendrogram and is only present in Ecological Site 1 (Figure 3). The reaches in this state (CH1, EP1, EP2, EP3, TU1, TU2, TU3, and UT1 in all years) generally had multitiered canopies of riparian trees: *Salix laevigata*,* Populus fremontii*,* Quercus lobata,* and the vine *Vitis californica*; and these clusters have many hydrophilic indicator species (Figure 4). Their close linkage distances in the cluster analysis as well as the ecological similarities of their indicator species suggest that changes between these plant community types may happen frequently and without major outside forcing. For that reason, the four clusters are included in the state‐and‐transition diagram as community phases within Vegetation State 1 (Figure 4).

#### 3.2.2 Vegetation state 2

Although most of the study reaches classified as Ecological Site 1 are clustered in a relatively cohesive area of the vegetation cluster dendrogram, one cluster (Cluster 3) is isolated from the rest of the Ecological Site 1 clusters (Figure 3). Unlike Vegetation State 1, vegetation in Cluster 3 is characterized by upland annual grasses and forbs in the herbaceous layer, and lacks any woody plant indicator species except the non‐native shrub *Nicotiana glauca*. None of the indicator species in Cluster 3 is considered hydrophilic (Figure 4). The study reaches that make up this cluster are the predominantly dry reaches: UT2 (all years), UT3 (all years), and CH2 (in 2015 and 2016 only). The generally dry conditions and ephemeral stream flow on UT2 and UT3 make it unlikely that these reaches will shift to a community dominated by woody plants and hydrophilic species characteristic of the other vegetation clusters in Ecological Site 1 without a major weather event and a subsequent change in geomorphology and hydrology. For that reason, Cluster 3 is included as a unique vegetation state (State 2) in the state‐and‐transition diagram (Figure 4).

#### 3.2.3 Vegetation state 3

In Ecological Site 2, there are three closely related vegetation clusters (clusters 2, 4, and 7). All three share a common branch of the cluster dendrogram (Figure 3) and share similar riparian shrub and tree species. The clusters have relatively high cover of *Salix goodingii*,* Populus fremontii,* and *Baccharis salicifolia*, relatively low cover of *Salix laevigata,* and no *Quercus lobata* cover. Given the similarities in perennial riparian vegetation and their proximity on the cluster dendrogram, these three vegetation clusters are all considered phases in Vegetation State 3 (Figure 4).

### 3.3 Transitions and phase shifts

Spatial variation within each of the ecological sites was more pronounced than temporal change over the study period. In total, seven community phases (i.e., the vegetation clusters) comprising three vegetation states were observed across the reaches in Ecological Site 1, and three community phases were observed across the reaches in Ecological Site 2 (Figure 4). Of all the potential “spatial transitions,” compelling evidence only exists for the cause of one transition in Ecological Site 1 between Vegetation State 2 and Vegetation State 1 (T2, Figure 4). In Ecological Site 1, one minor “temporal” phase shift and one more significant “temporal” transition were also observed over the 4 years of the study; and only one phase shift was observed on reaches in Ecological Site 2. A summary of these transitions and phase shifts is below:

#### 3.3.1 Transition from vegetation state 2 to vegetation state 1 (spatially‐observed) (T2‐Ecological Site 1)

This transition occurs in Ecological Site 1 when Vegetation State 2 (Vegetation Cluster 3—characterized by dry stream reaches dominated by upland annual grasses) changes to Vegetation State 1 (Vegetation Cluster 6—characterized by perennially wet stream reaches dominated by hydrophilic plant species) (Figure 4). Although these two states both occur on Upper Tejon Creek, they are separated by a large head cut and have drastically different channel morphologies and stream flows. Above the head cut, study reaches UT2 and UT3 are multichannel reaches with short distances between thalweg and historical floodplains (2.0 and 1.5 m respectively). On these reaches, water flows ephemerally and upland plants are the dominant vegetation. Below the head cut, UT1 is a single channel reach with a much larger distance between thalweg and historic floodplain (7.2 m). On this reach, water flows year‐round and vegetation is a mix of wetland plants.

#### 3.3.2 Transition from vegetation state 3 to vegetation state 2 (temporally‐observed) (T1‐Ecological Site 1)

**Figure 5:**
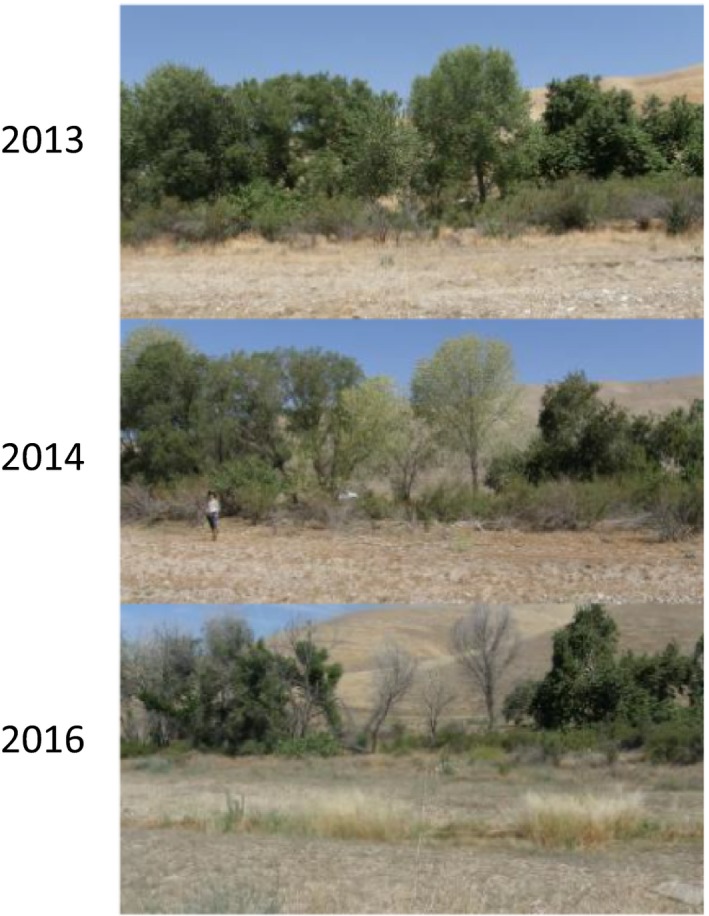
Photos of study reach CH2. Note the die‐back in tree canopy between 2013 and 2016. Vegetation on the reach was classified as Vegetation State 3 in 2013 and 2014, but changed to Vegetation State 2 in 2015 and 2016. There is no photo for 2015, but tree and shrub thinning was observed

In Ecological Site 1, the study reach CH2 changed from Vegetation State 3 (Vegetation Cluster 2—characterized by *Platanus racemosa* and *Baccharis salicifolia*) in 2013 and 2014 to the upland annual‐dominated Vegetation State 2 (Vegetation Cluster 3) in 2015 and 2016 (Figure 4). Although this transition occurred on a plot with a cattle exclosure, it is unlikely that the changes in cattle activity precipitated this change. The transition was characterized by a die‐off of established riparian trees and was more likely the result of 4 years of below‐average precipitation. During these years, *Populus fremontii* absolute cover in the tree canopy decreased from 40% in 2013 and 2014 to 5% in 2015 and 0% in 2016; *Salix laevigata* cover in the tree canopy decreased from 35% in 2013 to 13% in 2015 and 0% in 2016 (Figure 5, Figure S1). Reversing this transition may require several years of wet conditions to reestablish these tree species.

#### 3.3.3 Phase shift from vegetation cluster 1 to 6 (temporally‐observed) (PS1‐Ecological Site 1)

In Ecological Site 1, the study reach EP3 changed from Vegetation Cluster 1 to Vegetation Cluster 6 between 2015 and 2016 sampling. This represented a phase shift from a community dominated by *Vitis californica* and *Salix laevigata* in the herbaceous layer to one characterized by a suite of herbaceous hydrophilic plants (Figure 4). This phase shift followed an unusual summer flood in 2015 that cleared out some of the woody plant understory.

#### 3.3.4 Phase shift from vegetation cluster 7 to 2 (temporally‐observed) (PS1‐Ecological Site 2)

The only phase shift observed in Ecological Site 2 was when the study reach LT2 changed from Vegetation Cluster 7 to Vegetation Cluster 2 between 2013 and 2014 sampling. This represented a shift from a community characterized by *Salix goodingii*,* Populus fremontii*, and a suite of herbaceous hydrophytes to a community characterized by high cover of *Baccharis salicifolia* in the herbaceous canopy. The shift occurred after herbaceous hydrophyte and *Salix goodingii* cover decreased in 2014, possibly also the result of below‐average precipitation (Figure 4).

### 3.4 Results of CART analysis

**Figure 6:**
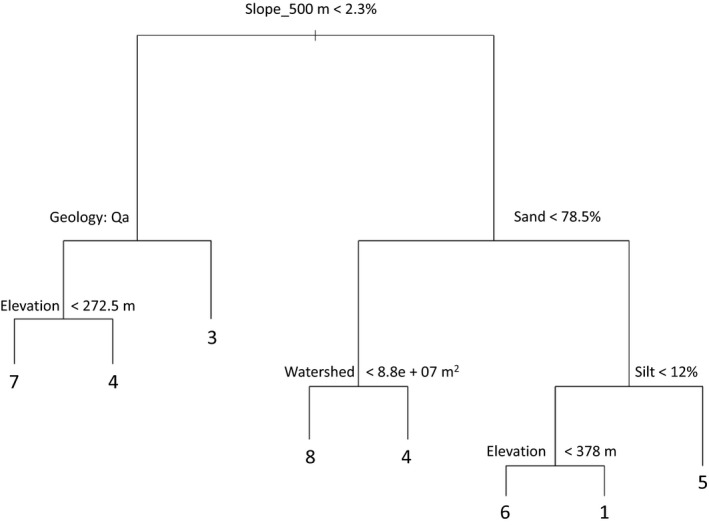
Results from a classification tree with vegetation clusters as the categorical response variable, and the abiotic Ecological Site variables, total annual precipitation, and cattle exclosures as the factors used to split the data. The splits farther up in the tree explain more of the overall variation in vegetation

The root split in the classification tree was channel slope (500 m), indicating that it explained the most variation in vegetation phases. After that, a combination of soil texture, geology, watershed size, and elevation were the factors chosen to further partition the cluster assignments. The reach‐scale stream geomorphological measurements, cattle exclosures, and annual precipitation were not included in the pruned classification tree, indicating that they did not consistently predict the different vegetation clusters (Figure 6). Overall, the pruned CART model correctly classified 90% of the Reach × Years, with only six Reach × Years misclassified.

## 4 DISCUSSION

The distribution of the vegetation states and phases was largely explained by the two ecological sites (Figures 3 and 4). Similarly, phases and states from each of the ecological sites occurred largely on separate branches of the classification tree (Figure 6), suggesting that ecological site characteristics corresponded closely with differences in vegetation. This coupling of ecological sites with vegetation phases and states is significant as it shows that the ecological site classification (based purely on abiotic variables) can be used to explain the occurrence of almost all of the vegetation phases on the study reaches and shows that each ecological site supported unique vegetation states.

The 15 study reaches are on the boundary of two floristic regions in California: (1) the San Joaquin Valley Subregion, and (2) the Tehachapi Mountain Area Subregion (Baldwin, 2012). They are also on the boundary of two Major Land Resource Areas (MLRAs). The higher elevation reaches in Ecological Site 1 are closer to the Tehachapi Subregion and fall in the Sierra Nevada Foothills MLRA, and the reaches in Ecological Site 2 fall squarely in the San Joaquin Valley Subregion and are closer to the Sacramento and San Joaquin Valley MLRA. While it is not surprising that the two ecological sites—which describe an elevation gradient on the boundary of the regions—have distinct vegetation communities, it is validating that they also detect differences in vegetation predicted by these biogeographic regions.

The classification tree highlighted which environmental factors corresponded to differences seen in the vegetation communities and gave a good indication of how best to adapt existing ecological site concepts to create meaningful predictions for riparian rangelands in this region. It showed the importance of the underlying site variables: channel slope, elevation, geology, watershed size and soil texture; suggesting that in this system—in addition to upland ecological site criteria—channel slope and contributory watershed size should be considered when classifying riparian ecological sites.

Surprisingly, with the exception of channel slope, stream geomorphological factors were not important in the classification tree. This implies that none of these geomorphological variables consistently predicted differences in the vegetation phases. Stream cross‐sectional profiles differed at large spatial scales, as seen in the differences between ecological sites (Table 2), but they also varied at relatively small spatial scales throughout the study area (e.g., between study reaches on a creek segment). Channel geomorphology was only measured once at each reach during the study; however, it was considered relatively stable over the study period because of the below‐average rainfall.

Cattle exclosure and precipitation were also not significant variables in the CART analysis. This makes sense given that (1) cattle exclosures were only in place for two years; (2) rainfall was not highly variable over the study period; and (3) cluster indicator species contained many perennial woody species. The apparent lack of influence from cattle grazing raises important questions for management of this system, including:

1. Does cattle activity affect vegetation states over longer periods of time? If so, what levels of sustained cattle activity result in qualitative changes to vegetation, and what are the primary mechanisms of change (e.g., hindering woody plant recruitment)? Are these changes contingent on ecological site?
2. Are certain states or phases more sensitive to cattle activity? In particular, are reaches with more herbaceous, perennial hydrophytes (e.g., Cluster 6 in Ecological Site 1) more likely to be affected by cattle?
3. What opportunities exist for enhancing or maintaining riparian vegetation? Are there interactions between grazing and rainfall or grazing and acorn mast years that should be taken advantage of or avoided? Does grazing management in Upper Tejon Creek affect the rate at which the head cut in that stream segment moves upstream—and thus the vegetation state? Could management strategies—such as seasonal grazing regimes, bank stabilization using restoration planting, or moderating peak stream discharge using an existing dam—slow or stop the movement of the head cut and prevent vegetation from transitioning from State 2 to State 1?

These questions can be formulated as formal hypotheses and tested through longer‐term monitoring of exclosures or riparian pastures with prescribed stocking rates. The ecological sites and vegetation states/phases identified in this study provide ecological context that can guide managers’ selection of study locations, treatments, and monitoring methods to efficiently answer these questions. Further investigation in this area will result in better descriptions of states, drivers of transitions, and ecological site boundaries. Future research should also investigate the distribution and extent of these ecological sites and their associated state‐and‐transition dynamics in the southern San Joaquin Valley and the Sierra Nevada Foothills MLRA.

## 5 CONCLUSIONS

By including riparian‐specific criteria, ecological site classifications can be built for riparian systems. On Tejon Ranch, riparian ecological site descriptions and state‐and‐transition models provided a unified framework linking abiotic and management factors to vegetation dynamics. These models were able to incorporate and organize highly variable riparian site factors and vegetation assemblages. By cataloging known phases, states, and transitions on each ecological site, these models created an organized approach to understanding the complex and site‐specific responses of rangeland riparian areas. They provided a framework for predicting vegetation states and transitions, and for generating and testing hypotheses linking weather, management, and site characteristics to vegetation changes over time and space.

## CONFLICT OF INTEREST

None declared.

## AUTHOR CONTRIBUTION

FR, JB, SS, and MW developed the ideas and methodology; FR, JB, SS, and MW collected the data; FR analyzed the data, with contributions from JB, LM, SS, and MW; FR led the writing of the manuscript with substantial contributions from JB, LM, SS, and MW. All authors gave final approval for publication.

## DATA ACCESSIBILITY

Data will be made available in the Dryad Digital Repository.
