# Supplementary material for: Applying ecological site concepts and state‐and‐transition models to a grazed riparian rangeland
Source: Ecol Evol. 2018 Apr 19;8(10):4907–18. doi: 10.1002/ece3.4057 (PMC5980404; doi:10.1002/ece3.4057)

Supporting Information:

**Supporting Table 1.** Species recorded on greenline transects on the study reaches. The table contains attributes of each species.

| **Scientific name^1^** | **Common name** | **Origin** | **Annual/ Perennial^2^** | **Functional group** | **Family** | **Wetland Status^4^** | **Congener Status** |
| --- | --- | --- | --- | --- | --- | --- | --- |
| *Anemopsis californica* | Yerba Mansa | native | perennial | forb | Sauraceae | OBL |  |
| *Apium graveolens* | Celery | exotic | Annual/ biennial | forb | Apiaceae |  |  |
| *Artemisia douglasiana* | Mugwort | native | perennial | forb/shrub | Asteraceae | FAC |  |
| *Avena barbata* | Slender Wild Oats | exotic | annual | grass | Poaceae |  | UPL |
| *Baccharis salicifolia* | Mule Fat | native | perennial | shrub | Asteraceae | FAC |  |
| *Berula erecta* | Water parsnip | native | perennial | forb | Apiaceae | OBL |  |
| *Brassica nigra* | Black Mustard | exotic | annual | forb | Brassicaceae |  | FACU |
| *Bromus diandrus* | Ripgut Brome | exotic | annual | grass | Poaceae |  | FACU or UPL |
| *Bromus hordeaceus* | Soft Chess | exotic | annual | grass | Poaceae | FACU |  |
| *Bromus madritensis* | Foxtail Brome | exotic | annual | grass | Poaceae | UPL |  |
| *Bromus rubens* | Red Brome | exotic | annual | grass | Poaceae |  | FACU or UPL |
| *Bromus tectorum* | Cheatgrass | exotic | annual | grass | Poaceae |  | FACU or UPL |
| *Carduus pycnocephalus* | Italian Thistle | exotic | annual | forb | Asteraceae |  | FACU |
| *Cynodon dactylon* | Bermuda Grass | exotic | perennial | grass | Poaceae | FACU |  |
| *Distichlis spicata* | Saltgrass | native | perennial | grass | Poaceae | FAC |  |
| *Eleocharis parishii* | Parish's Spikerush | native | perennial | graminoid | Cyperaceae | FACW |  |
| *Eleocharis sp.* | spike rush | native | perennial | graminoid | Cyperaceae |  | FACW or OBL |
| *Festuca myuros* | Foxtail Fescue | exotic | annual | grass | Poaceae | FACU | FACU |
| *Festuca perennis* | Italian rye grass | exotic | perennial | grass | Poaceae | FAC | FAC |
| *Galium aparine* | Common Bedstraw | native | annual | forb | Rubiaceae | FACU |  |
| *Gnaphalium sp.* | cudweed | native | annual/ perennial | forb | Asteraceae |  |  |
| *Hirschfeldia incana* | Shortpod Mustard | exotic | perennial | forb | Brassicaceae |  |  |
| *Hordeum murinum* | Foxtail Barley | exotic | annual | grass | Poaceae | FACU |  |
| *Juncus bufonius* | Common Toad Rush | native | annual | graminoid | Juncaceae | FACW |  |
| *Juncus mexicanus* | Mexican Rush | native | perennial | graminoid | Juncaceae | FACW |  |
| *Juncus sp.* | Rush | unknown | unknown | graminoid | Juncaceae |  | FACW or OBL |
| *Juncus xiphioides* | iris leaved rush | native | perennial | graminoid | Juncaceae | OBL |  |
| *Lactuca serriola* | Prickly Lettuce | exotic | annual | forb | Asteraceae | FACU |  |
| *Lemna sp.* | Duckweed | native | perennial | forb | Araceae |  | OBL |
| *Medicago polymorpha* | Bur Clover | exotic | annual | forb | Fabaceae | FACU |  |
| *Melilotus indicus* | Annual Yellow Sweetclover | exotic | annual | forb | Fabaceae | FACU |  |
| *Mimulus guttatus* | Common Monkeyflower | native | perennial | forb | Phrymaceae | OBL |  |
| *Nasturtium officinale* | Watercress | native | perennial | forb | Brassicaceae | OBL |  |
| *Nicotiana glauca* | tree tobacco | exotic | perennial | shrub | Solanaceae | FAC |  |
| *Persicaria lapathifolia* | Common knotweed | native | annual | forb | Polygonaceae | FACW |  |
| *Platanus racemosa* | California Sycamore | native | perennial | tree | Platanaceae | FAC |  |
| *Poa annua* | Annual Bluegrass | exotic | annual | grass | Poaceae | FAC |  |
| *Polypogon monspeliensis* | Annual Beard Grass | exotic | annual | grass | Poaceae | FACW |  |
| *Polypogon sp.* | Beard Grass | exotic | unknown | grass | Poaceae |  | FACW |
| *Polypogon viridis* | Water beard grass | exotic | perennial | grass | Poaceae | FACW |  |
| *Populus fremontii* | Fremont cottonwood | native | perennial | tree | Salicaceae |  | FACW, FAC, or FACU |
| *Quercus lobata* | Valley oak | native | perennial | tree | Fagaceae | FACU |  |
| *Rumex sp.* | dock | unknown | unknown | forb | Polygonaceae |  | FACW, FAC, FACU |
| *Salix gooddingii* | Goodding's black willow | native | perennial | tree | Salicaceae | FACW |  |
| *Salix laevigata* | Red Willow | native | perennial | tree | Salicaceae | FACW |  |
| *Salix lasiolepis* | Arroyo willow | native | perennial | tree | Salicaceae | FACW |  |
| *Silybum marianum* | Milk thistle | exotic | annual/ perennial | forb | Asteraceae |  |  |
| *Sonchus sp.* | Sow Thistle | exotic | annual/ perennial | forb | Asteraceae |  | FAC or FACU |
| *Stachys sp.* | Hedge Nettle | native | perennial | forb | Lamiaceae |  | OBL or FACW |
| *Stellaria media* | Chickweed | exotic | annual | forb | Caryophyllaceae | FACU |  |
| *Tamarix ramosissima* | Saltcedar | exotic | perennial | tree | Tamaricaceae |  | FAC |
| *Torilis arvensis* | Hedge Parsley | exotic | annual | forb | Apiaceae |  |  |
| *Urtica dioica* | Stinging nettle | native | perennial | forb/shrub | Urticaceae | FAC |  |
| *Urtica urens* | Annual Stinging Nettle | exotic | annual | forb | Urticaceae |  |  |
| *Veronica anagallis-aquatica* | water speedwell | exotic | perennial | forb | Plantaginaceae | OBL |  |
| *Vitis californica* | California grape | native | perennial | vine | Vitaceae | FACU |  |
| *Xanthium strumarium* | Cocklebur | native | annual | forb | Asteraceae | FAC |  |

Scientific names consistent with “The Jepson Manual” (Baldwin 2012)

Annual/Perennial information taken from Calflora. <http://www.calflora.org>

Cal-IPC ratings taken from California Invasive Plant Council “California Invasive Plant Inventory Database” <http://www.cal-ipc.org/paf/>

Wetland status taken from the Army Corps of Engineers “National Wetland Plant List” (Lichvar et al. 2016) <http://rsgisias.crrel.usace.army.mil/NWPL/> The wetland plant codes are:

OBL = Obligate Wetland

FACW = Facultative Wetland

FAC = Facultative

FACU = Facultative Upland

UPL = Upland

**References**

Baldwin, B.G. (Ed.), 2012. The Jepson manual: vascular plants of California, 2nd ed. ed. University of California Press, Berkeley, Calif.

Lichvar, R.W., Banks, D.W., Kirchner, W.N., Melvin, N.C., 2016. The National Wetland Plant List: 2016 wetland ratings. Phytoneuron 30, 1–17.

**Supporting Figure 1.** Percent cover in each canopy layer at CH2. The study reach changed from Vegetation State 3 (characterized by *Platanus racemosa* and *Baccharis salicifolia*) in 2013 and 2014 to the upland annual-dominated Vegetation State 2 in 2015 and 2016.


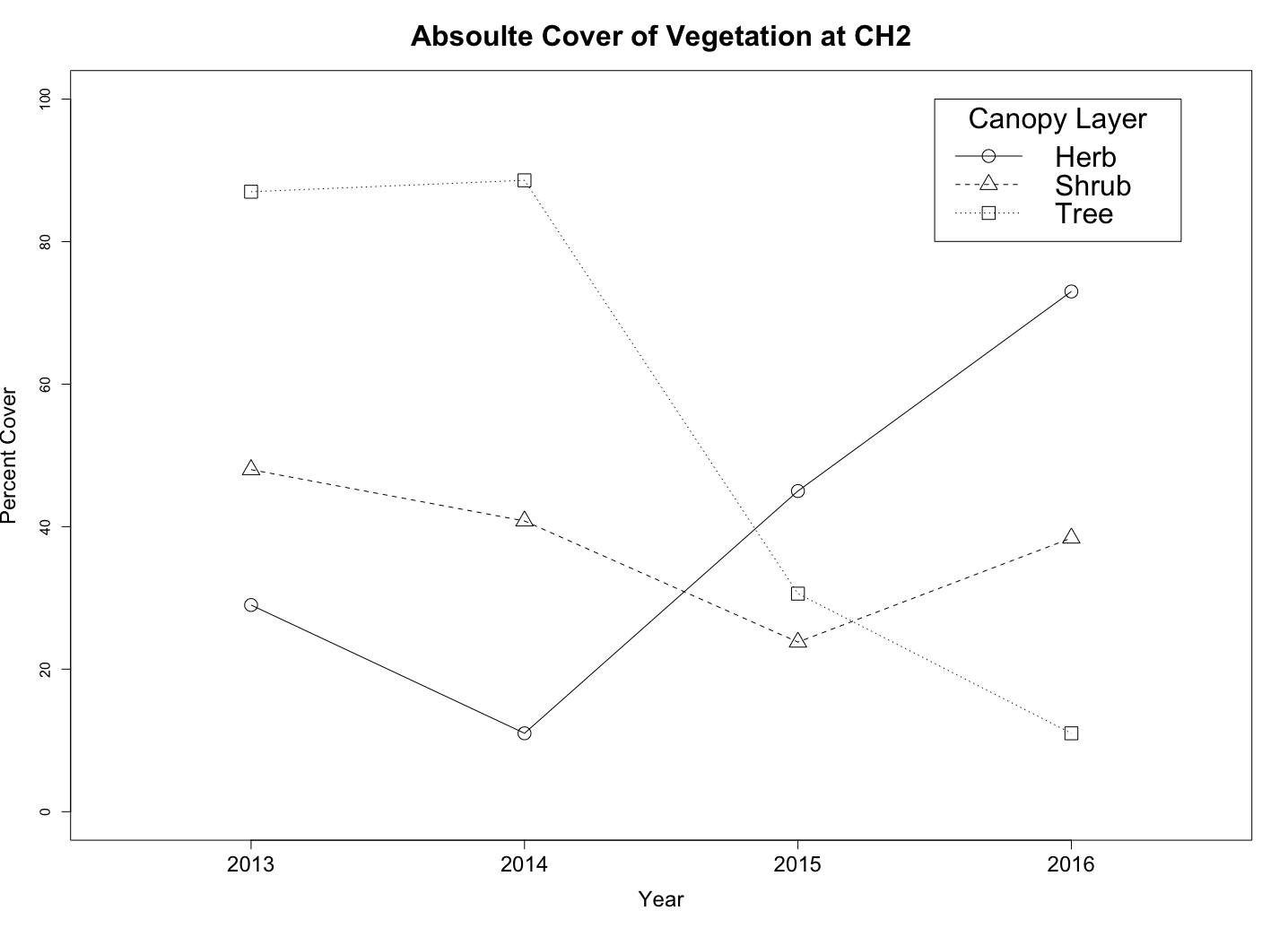

Supplement: Supplementary file 1 [file ECE3-8-4907-s001.docx]
